# Bedside direct observation of medical student-performed physical examination is highly rated in student satisfaction

**DOI:** 10.15694/mep.2017.000021

**Published:** 2017-02-01

**Authors:** Jacquelyne Cios, J. Chad Hoyle, Adam Quick, Nicole V. Brown, Curtis Walker, Jr., Kimberly Tartaglia

**Affiliations:** 1 Ohio State University College of Medicine; 2 Ohio State University Department of Statistics

**Keywords:** Bedside teaching, Physical examination, Student satisfaction, Neurology, Direct observation

## Abstract

This article was migrated. The article was marked as recommended.

**Objectives:** To evaluate satisfaction among third year medical students with a bedside teaching exercise comprised of direct observation of student-performed physical examination skills and related feedback.

**Methods:** An observational, cross-sectional study design was employed to study third year medical students undergoing the Neurology clerkship at the Ohio State University College of Medicine between June and October 2015. Immediately following the bedside physical examination teaching exercise, student satisfaction data was obtained in anonymous survey (n=21). In addition, student satisfaction data from the class cohort (n=51), regarding various learning formats in the curriculum, were collected at the end of a 16-week block of rotations including the Neurology clerkship. Data were summarized using descriptive statistics.

**Results:** Most students felt that their level of confidence increased as a result (85.0%, n=17/21), and they felt they would use what they had learned in the future (95%, n=19/21). Only about half of the students felt strongly that reflection on the learning experience was sought (47.6%, n= 10/21). At the end of the 16 weeks block, the Neurology examination exercise was rated among the most highly in student satisfaction (3.35/4, SD=0.89) as compared to procedural workshops (2.76/4, SD= 0.76), other small group topic format (2.78/4, SD= 0.85), and traditional lecture (2.39/ 4, SD= 0.89).

**Conclusions:** The bedside direct observation of physical examination performed by medical students is highly rated in student satisfaction, and students are most satisfied with this format of teaching among all formats studied. Increased opportunity for reflection in this setting represents an area for further development.

## Background

Feedback has been considered an integral part of effective practice in medical education. ^1^ It has been observed that feedback which is improperly given can result in unwanted consequences at times far into the trainee's career. ^2^

A recent review of assessment tools in medical education demonstrated that structured direct observation activities play an important role in student medical education. ^3 ^In addition, this idea has been reinforced by the results of a systematic review of the effectiveness of continuing medical education strategies. The study, which examined 99 randomized controlled trials of strategies that assessed physician performance, concluded that high impact interventions include practice-based interventions rather than formal audits, educational materials, or conferences and lectures. ^4 ^While direct observation is in limited use given its cost and time-consuming nature, it has been observed to impact students' future learning and practice. Both students and educators tend to accept this method of learning by offering individualized feedback by credible experts. Learners may be empowered by this personal attention as well as the realization of their own capability in light of this close partnership. For these reasons, the Lead.Serve.Inspire curriculum of the Ohio State University College of Medicine has implemented structured direct observation of students performing the physical exam. In the third year of the curriculum, this is built into the Neurology clerkship and applied to the teaching of the neurologic physical examination.

Challenges face the medical educator regarding identification of the optimal manner, timing and quantity of feedback offered to the student, particularly during the teaching of physical exam skills in direct observation activities. Advantages and pitfalls are associated with using both real and standardized patients, such as for example, whether to pause the student's encounter to give feedback in real time, or to give the feedback after the entire physical examination is completed. The manner of feedback can vary widely, ranging from subtle reinforcement or modelling desired behavior to explicit instruction. It is unclear which methods or techniques for offering feedback during direct observation of physical exam are the most favorable. In addition, the educator is faced with the choice of type of feedback to give in terms of content and delivery style.

Feedback which successfully results in educational benefit has been identified to focus on observable behaviors rather than on the personality or other attributes of the trainee. ^5^ In addition, Hewson et al. conducted a faculty development activity which polled a range of clinician educators from various disciplines and assessed which feedback techniques were successful, producing a list of nine different recommended aspects of effective feedback or educator behaviors (Table 1). ^6^

Table 1. Nine characteristics of productive feedback in medical education. 

**Table:** 

Teacher establishes appropriate interpersonal climate
Feedback given in appropriate location
Feedback given regarding mutual teacher- learner goals
Teacher elicits learner's thoughts/ feelings
Reflection on behavior is elicited
Feedback is nonjudgmental
Right amount of feedback is given
Feedback is specific
Ideas for improvement are suggested

Given this framework, the purpose of this study is to characterize the types of feedback given by two different neurologist clinician-educators during the third-year medical student direct observation activity at the Ohio State University and determine the anonymously self-rated level of student satisfaction with the direct observation exercise, among a sample of third year Neurology medical students. In addition, the student-rated satisfaction with this format of learning compared to other small-group learning formats and traditional lecture is examined.

## Methods

All neurology clerkship students, between June and October 2015, were assigned with one of the study investigators to undergo the direct observation assignment, for the purpose of receiving formative feedback regarding their observed neurologic physical examination. All feedback was collected anonymously using a questionnaire analyzing Hewson's aspects of feedback, and a satisfaction survey. 

The observation feedback questionnaire was a template on which to track the types of feedback given, specified according to nine categories as modeled in Table 1. Each student rated on a Likert scale of 1-5 to what degree the feedback they received fulfilled each of the categories specified in Table 1. The second form was a satisfaction survey regarding the student's satisfaction with the feedback provided in the direct observation exercise, and was completed by the learner (Table 2). In addition, some demographic information such as age and gender of the student, how well the student knew the classmates within their small group, the timing and location of this feedback, and the name of the educator, was recorded.

The neurology clerkship experience, of which the direct observation exercise is a small part, represents in turn a small portion of the integrated "Patients with Specialized Medical Needs (SMN) Component of the curriculum, which is termed the "SMN ring." This includes a combination of experiences in Internal Medicine, Psychiatry and Neurology, and data regarding student satisfaction for the ring as a whole (n=61) are collected. This cohort includes the 20 students who completed the survey and questionnaire. 

Descriptive statistics were used to summarize all survey questions and observation feedback forms. Categorical responses were expressed using frequencies and percentages. Descriptive statistics were also used to describe results of student satisfaction with various components of the SMN ring, after conclusion of the ring, including but not limited to the Direct Observation examination teaching exercise.

## Results

A total of 20 / 27 (74.1 %) students responded to both the observation feedback questionnaire and the satisfaction survey. Seven students responded to the observation feedback questionnaire using an outdated version of the form and were excluded from the analysis. One additional student responded only to the characterization questionnaire.

Satisfaction Survey: 

Among the 20 students who completed the satisfaction surveys, 9 (56.2%) were female and 7 (43.8%) were male (4 students did not specify gender). The average age of students was 25.2 years (SD=1.8, range from 22 to 29, n=15; 5 students did not specify age). 

Most students who responded felt that their goals had been met by the exercise (100%, n=19), that their level of confidence in performing the neurologic examination increased as a result (85.0%), and they felt they would use what they had learned in the future (95%). 

When asked to rank how satisfied they felt with this exercise the majority of students reported at least some level of satisfaction, and 18 (90%) reported feeling somewhat or very satisfied. 

When comparing level of interest (somewhat interested or very interested) in neurology/neuroscience before and after the exercise, there was an overall increase going from 50% to 70% based on those that answered the question (Table 2).

Observation Feedback Questionnaire:

Among the 21 students who completed the observation feedback questionnaire, 8 (53.3%) were female and 7 (46.7%) were male (6 students did not specify gender). The average age was approximately 25.3 years (SD=1.8, range from 22 to 29, n=15). 

Most students thought that the teacher established appropriate interpersonal climate (85.7%), feedback given was in an appropriate location (76.2%), and feedback was nonjudgmental (90.5%). A moderate proportion of students felt that the right amount of feedback was given (66.7%), and that the feedback was specific (66.7%). Relatively lower scores on the feedback were given regarding successfully addressing mutual teacher-learner goals (57.1%) and elicitation of reflection (47.6%). 

Most feedback was given both during and after the exercise (80.0%) (Table 3).

Table 2. Neurology medical student satisfaction survey results after a teaching exercise involving direct observation of physical examination skills. 

**Table:** 

***Survey Question***	***Level***	Total (N=21*)
***Learner Responses***
How well do you know the other members of your group?1-Not at all, 5-A lot	*3*	6 (37.5%)
*4*	8 (50.0%)
*5*	2 (12.5%)
*Missing*	5
Level of interest in neurology/neuroscience PRIOR to completing this exercise	*Somewhat Uninterested*	4 (20.0%)
*Neutral*	6 (30.0%)
*Somewhat Interested*	9 (45.0%)
*Very Interested*	1 (5.0%)
*Missing*	1
How satisfied you felt with this exercise	*Very Dissatisfied*	1 (5.0%)
*Somewhat Dissatisfied*	1 (5.0%)
*Somewhat Satisfied*	5 (25.0%)
*Very Satisfied*	13 (65.0%)
*Missing*	1
My goals were met by this exercise*	*TRUE*	19 (100%)
*Missing*	2
My level of confidence in the neurologic physical examination increased as a result of this exercise	*TRUE*	17 (85.0%)
*FALSE*	3 (15.0%)
*Missing*	1
If you answered true, please rank the degree of impact the exercise made on your confidence level1-Not very much, 5-Very much	*2*	1 (5.9%)
*3*	6 (35.3%)
*4*	7 (41.2%)
*5*	3 (17.7%)
I learned some aspects of the neurologic examination as a result of this exercise that I did not learn during the course of participation in my clerkship activities	*TRUE*	17 (85.0%)
*FALSE*	3 (15.0%)
*Missing*	1
In the future, I will use aspects of the neurologic physical examination that I learned today	*TRUE*	19 (95.0%)
*FALSE*	1 (5.0%)
*Missing*	1
I found the one-on-one nature of the direct observation and the question and feedback opportunity linked with that to be beneficial1-Not very much, 5-Very much	*3*	3 (15.0%)
*4*	6 (30.0%)
*Very Much*	11 (55.0%)
*Missing*	1
Please indicate your level of interest in neurology/neuroscience AFTER completing this exercise	*Somewhat Uninterested*	3 (15.0%)
*Neutral*	3 (15.0%)
*Somewhat Interested*	12 (60.0%)
*Very Interested*	2 (10.0%)
*Missing*	1
If you felt dissatisfied with any aspect of the exercise, please specify	*Missing*	21

*includes one student who responded only to the observation feedback questionnaire.

Table 3.Neurology medical student observation feedback questionnaire results after a teaching exercise involving direct observation of physical examination skills.

**Table:** 

***Observation Feedback***	***Level***	***Total (N=21)***
Teacher establishes appropriate interpersonal climate:1-Extreme no, 5-Exteme yes	*4*	3 (14.3%)
*5*	18 (85.7%)
Feedback given in appropriate location1-Extreme no, 5-Exteme yes	*4*	5 (23.8%)
*5*	16 (76.2%)
Feedback given regarding mutual teacher-learner goals1-Extreme no, 5-Exteme yes	*3*	1 (4.8%)
*4*	8 (38.1%)
*5*	12 (57.1%)
Teacher elicits learners thoughts/feelings1-Extreme no, 5-Exteme yes	*3*	5 (23.8%)
*4*	4 (19.1%)
*5*	12 (57.1%)
Reflection on behavior is elicited1-Extreme no, 5-Exteme yes	*3*	5 (23.8%)
*4*	6 (28.6%)
*5*	10 (47.6%)
Feedback is nonjudgmental1-Extreme no, 5-Exteme yes	*4*	2 (9.5%)
*5*	19 (90.5%)
Right amount of feedback is given1-Extreme no, 5-Exteme yes	*2*	1 (4.8%)
*4*	6 (28.6%)
*5*	14 (66.7%)
Feedback is specific1-Extreme no, 5-Exteme yes	*3*	3 (14.3%)
*4*	4 (19.1%)
*5*	14 (66.7%)
Ideas for improvement are suggested1-Extreme no, 5-Exteme yes	*3*	3 (15.0%)
*4*	6 (30.0%)
*5*	11 (55.0%)
*Missing*	1 (0%)
Was the feedback given during the exercise, after the exercise or both?	*Both*	16 (80.0%)
*During*	3 (15.0%)
*Other	1 (5.0%)
*Missing*	1 (0%)
Order of patient seen (if you are first in a group of 5 students for the day, put 1; if you are second to do the exercise that day, put a 2, etc.):	*1*	5 (29.4%)
*2*	3 (17.7%)
*3*	4 (23.5%)
*4*	3 (17.7%)
*5*	2 (11.8%)
*Missing*	4 (0%)
Is this a patient who is familiar to you?	*No*	17 (94.4%)
*Yes*	1 (5.6%)
*Missing*	3 (0%)
Rate the level of difficulty of your patient case compared to that of the others in your group 5-Extreme difficulty	*1*	2 (11.1%)
*2*	2 (11.1%)
*3*	6 (33.3%)
*4*	6 (33.3%)
*5*	2 (11.1%)
*Missing*	*3*

*Other=Some during, some on the written form evaluation given after the end of the encounter

Student Satisfaction Assessment During the Integrated Internal Medicine-Neurology-Psychiatry (SMN) Ring Curriculum:

Satisfaction scores from 61 third year medical students were collected at the end of the Integrated Internal Medicine- Neurology- Psychiatry 16 week block, or "SMN ring." This group of students includes some of those that were participants in the direct observation exercise and completed the satisfaction survey and observation feedback questionnaire. Of these students, 30 (49.2%) were from the graduate class of 2016 while 31 (50.8%) were from the graduate class of 2017.

Of the 61 students in the integrated SMN curricular ring during the study period, 51 students anonymously rated the Neurology direct observation exercise, as part of a longer survey, at the end of the 16 week portion of the curriculum. A total of six faculty were assigned to lead these Neurology direct observation exercises, although the students of only two were included in the sample given the questionnaire and survey. The students rated their satisfaction on a scale of 0-4, with 4 being the highest degree of satisfaction, equivalent to "excellent" rating, and 0 indicating a "non-applicable" response (Table 4). 

Table 4. Student satisfaction ratings (n=51) during the Specialized Medical Needs (SMN) Ring, the integrated Internal Medicine, Neurology, and Psychiatry third year medical student clinical experiences. Ratings were given upon completion of the 16 week portion of the curriculum, which included the Neurology Direct Observation physical examination assignment, and all were based on a scale of 0-4 (with 4 as the highest rating).

**Table:** 

Course Component	AverageRating	MinimumRating	Maximum Rating	Non-zero responses	Std Dev
Formal didactic session (case discussions, lectures)	2.39	1	4	51	0.94
Medicine mentors (small groups, no direct observation physical examination task)	2.67	1	4	51	1.14
Procedural workshops	2.76	1	4	51	0.76
Select Topic small groups	2.78	1	4	49	0.85
Student Report small groups	2.78	1	4	50	0.79
Direct observation physical examination by a Medicine Mentor	3.02	1	4	51	0.97
Direct observation of neurology exam by assigned faculty	3.35	1	4	51	0.89
GI exam rounds	3.56	2	4	50	0.58
Cardiology exam rounds	3.58	2	4	45	0.66

## Discussion

Medical education in physical examination skills continues to represent the keystone of quality instruction in the diagnostic method. Yet, in a recent study, Haring et al. found that approximately 40% of the core internal medicine physical examination was not performed or not performed satisfactorily by a cohort of medical students who had just completed the Internal Medicine clerkship. ^7^ The authors further point out the good intra-class correlation seen in their data set. This type of data for Neurology medical students is not available, but this may be concerning if the data is similar regarding the relatively small percentage of the core neurologic examination that is able to be performed by the majority of the class. Direct observation of a students' physical examination skills can play an important role in investigating and improving this.

Other medical centers' training programs have implemented direct observation programs and have shown a measurable increase in student reported rates of the performance of direct observation, reporting improvement of baseline indices to 82- 96%. ^8^ The fundamental purpose of this is to ensure that classic physical findings or cases have been observed and formative feedback is given, and this is a widely agreed to be a necessary part of medical education. 

At the Ohio State University, Neurology is one of the first departments to formalize the nature of physical examination teaching and establish a small group teaching climate with personalized instruction, through the direct observation exercise. This initial data shows that there are many aspects of the exercise that are successful from the viewpoint of the learner, including eliciting of mutual goals, eliciting of learner thoughts and feelings, and the offering of the "right amount" of specific feedback. However, reflection on the learning experience is one element that was relatively absent.

These data regarding the satisfaction among the entire cohort of 51 students undergoing the SMN ring concurrently show a high degree of satisfaction with the Neurology direct observation exercise. This correlates with our findings among the students in the smaller direct observation sample and suggests a high degree of interrater reliability among Neurology faculty in the quality of feedback given. In addition, in some cases this SMN rating occurred 15 weeks following the Neurology direct observation exercise, and therefore the consistency of the satisfaction suggests persistence of the satisfaction over time and with slightly greater clinical and academic experience.

Interestingly, when comparing the student satisfaction between small group activities that involve direct observation versus small group activities that do not involve this type of instruction, students clearly prefer the activities that involve contact with patients and direct observation of the student-patient and/or doctor-patient interaction. The students rated small group sessions with an Internal Medicine faculty mentor 2.67 on a 0-4 scale (SD= 1.14) when direct observation of exam was not an included element, but they rated it 3.02 (SD= 0.97) with the direct observation component. The students rated working with ring faculty in various select topics a 2.78 (SD= 0.85). However, Cardiology Exam rounds earned an average rating of 3.58 (SD= 0.66) and Gastroenterology Exam rounds earned an average rating of 3.58 (SD= 0.58) (Table 3). This suggests that the small group setting itself is not the essential element to highly satisfying learning forums. The elements common to the highest scoring educational tasks are the direct observation by attending faculty of student performance and immediate feedback. Therefore, third year medical students do not simply prefer a learning environment with a small student-faculty ratio. Specifically, they prefer the small group learning style where they are shown in a hands-on way how the study of pathology and anatomy is brought together with the clinical disciplines to result in a meaningful outcome, and this did not appear to be limited to the Neurology experience. 

It should be noted that the satisfaction rating pertaining to lectures, presented in this study, applies to the learners' satisfaction with the teaching method of formal lectures rather than the content or style of the individual lectures. These individual lectures when rated by students on their quality and delivery rank above average. However, our data show that the experience of the lecture is not as popular as that of the direct observation exercise. 

Further, this data helps to define the scope of our teaching practice, and future projects may use video in teaching exercises to illustrate multiple specific methods of giving successful feedback. In addition, this study will help us to design future studies, aimed at testing specific interventions including testing the direct observation educational task with pre- and post- assessments, and possibly directly correlating it with student objective performance such as test and quiz performance. Finally, serial performance of direct observation exercises, culminating in a summative exercise, may be of further benefit ^9^ and has not been studied with correlation to objective performance measures.

We further hope to identify trends regarding characteristics of feedback that are well-received by the learner and which may produce measurable improvement in performance, building upon the current understanding of the aspects of successful feedback in medical education. A previous pilot study done by the authors ^10^, suggested that among the most important aspects of learner feedback is the opportunity for self-reflection. Almost half of responders in this study agreed strongly that this opportunity for reflection was given, and further improvement could be made on this front. Of note, other items ranked on the questionnaire likely overlap with reflection on learning, including the eliciting of the learner's thoughts and feelings and the establishment of appropriate interpersonal climate.

Regarding opportunity for reflection, the implementation of serial skills measurement would be expected to be beneficial. There is a role for repeating a direct observation toward the end of the rotation or prior to graduation, using it as a summative or evaluative assessment. Reflection upon the hypothetical improvement between formative or first direct observation exercise and the follow-up exercise and how it was achieved would undoubtedly be satisfying for both educator and learner.

Wiener and Nathanson have described a schematic for classifying the types of errors learners make in physical examination, codifying them as errors of technique, omission, detection, interpretation and recording. ^11^ In particular, the most common errors they found in the neurologic examination in their study of 145 students and residents included technical errors regarding visual field testing, reflex testing, detection of mild paresis and ocular defects, to name a few. These are the some of the same of the same errors that we observed as well, and further investigation of type of student error, as well as the best technique for remediating it, is an area of future interest. 

A potential limitation is that the sample size for the survey and questionnaire is small. Further, there is possible response bias for students who did not respond and who may have felt differently about the exercise, as compared to those who did respond. In addition, 18 of the students completed their exercise with one of the investigators and 3 of them with another. Given the small number of participants who completed the exercise with the second educator, a difference between the two educators' techniques could not be assessed.

Another limitation of the study is the lack of objective academic performance-related correlation, which is inherent to the anonymous nature of the feedback data collection. Since the satisfaction was measured anonymously, objective measures of performance, such as Objective Structured Clinical Examination (OSCE) scores, could not be correlated with the satisfaction data for each student. The desired correlation between certain types of feedback given and academic performance assessing, for example, localization aptitude and interpretation of a set of findings, can be obtained by collecting data non-anonymously. This represents an area of future study.

The concept of neurophobia, ^12^ or a fear of the neurosciences by both medical students and non-neurologists has been described, and there is a need for improved clinical instruction in this area. The method of structured direct observation of physical examination is a good clinical teaching model and deserves further study.

## Take Home Messages

- Bedside direct observation of medical student performance of physical examinations scores highly in student satisfaction.
- Of all learning formats studied, bedside direct observation of student performance of physical examination skills followed by corrections and feedback from faculty scored more highly than any other format, including small group exercises of similar teacher: student ratio, and far more highly than traditional lectures.
- Use of the bedside direct observation teaching and learning format as serial exercise, both for formative and summative purposes, deserves further study, particularly for examination technique-heavy medical specialties, such as Neurology.

## Notes On Contributors

Jacquelyne Cios, MD MS is a neurologist and Expert Educator at the Ohio State University College of Medicine.

J. Chad Hoyle, MD is a neurologist and Understanding Patients with Specialized Medical Needs (Integrated Internal Medicine, Neurology, and Psychiatry Clerkships) "ring" director at the Ohio State University College of Medicine.

Adam Quick, MD is a neurologist and Neurology Clerkship director at the Ohio State University College of Medicine.

Nicole V. Brown is a biostatistician at the Ohio State University.

Curtis Walker, Jr, PhD is an education researcher in the Office of Evaluation, Curriculum Research, and Development at the Ohio State University College of Medicine.

Kimberly Tartaglia, MD is an internist and Director of the Third Year medical curriculum at the Ohio State University College of Medicine.

## Bibliography/References

1. Issenberg SB, McGaghie WC, Petrusa ER, Gordon DL, Scalese RJ. Features and uses of high fidelity medical simulations that lead to effective learning: a BEME systematic review. Medical teacher. 2005; 27(1): 10-28.

https://doi.org/10.1080/01421590500046924

2. Ende J. Feedback in clinical medical education. JAMA. 1983; 250:777-81.

https://doi.org/10.1001/jama.1983.03340060055026

3. Epstein RM. Assessment in medical education. New England Journal of Medicine. 2007; 356: 387-396.

https://doi.org/10.1056/NEJMra054784

4. Davis D, Thomson MA, Oxman A, Haynes RB. Changing physician performance: a systematic review of the effect of continuing medical education strategies. JAMA. 1995; 274(9): 700-705.

https://doi.org/10.1001/jama.1995.03530090032018

5. Ende, J. Feedback in clinical medical education. JAMA. 1983; 250:777-81.

https://doi.org/10.1001/jama.1983.03340060055026

6. Hewson MG, Little ML. Giving feedback in medical education: verification of recommended techniques. Journal of General Internal Medicine. 1998; 13(2): 111-116.

https://doi.org/10.1046/j.1525-1497.1998.00027.x

7. Haring CM, Cools, BM, van der Meer JWM, Postma CT. Student performance of the general physical examination in internal medicine: an observational study. BMC Medical education. 2014;14: 73.

https://doi.org/10.1186/1472-6920-14-73

8. Schiller J, Hammond M, Belmonte D, Englesbe M, Gelb D, Grum C, Heidelbaugh J, House J, Weir S, Santen S. Systematic Direct Observation of Clinical Skills in the Clinical Year. MedEdPORTAL Publications [serial online]. 2014 [cited 2016 July 15]. Available from: https://www.mededportal.org/publication/9712. 

9. Wiener, S, Nathanson, M. Physical examination: frequently observed errors. JAMA. 1976; 236 (7): 852-855.

https://doi.org/10.1001/jama.1976.03270080034028

10. Cios J, Hoyle JC, Quick, A. Assessment of the Characteristics of Formative Feedback in a Neurologic Physical Examination Direct Observation Exercise in Third Year Medical Students. Poster session presented at: the 2015 Central Group on Educational Affairs, Spring Meeting; 2015 June 23; Columbus, Ohio. Available from: https://www.mededportal.org/icollaborative/resource/4031. 

11. Wiener, S, Nathanson, M. Physical examination: frequently observed errors. JAMA. 1976; 236 (7): 852-855.12.Matthias, AT, Nagasinha P, Ranasinghe P, Gunatilake SB. Neurophobia among medical students and non-specialist doctors in Sri Lanka. BMC Medical Education. 2013;13:164.

https://doi.org/10.1186/1472-6920-13-164

## Appendices

## Declarations

The author has declared that there are no conflicts of interest.

