# Supplementary material for: Bedside direct observation of medical student-performed physical examination is highly rated in student satisfaction
Source: MedEdPublish (2016). 2017 Feb 1;6:21. [Version 1] doi: 10.15694/mep.2017.000021 (PMC10885231; doi:10.15694/mep.2017.000021)
Supplement: Peer review history

# Reviewer report: Reviewer response for version 1

**DOI:** 10.21956/mep.19436.r28595

**Published:** 2020-12-20

**Authors:** Megan Anakin

**Affiliations:** 1 University of Otago

This review has been migrated. The reviewer awarded 2 stars out of 5 https://www.mededpublish.org/manuscripts/821I read your article with interest because at my medical school, we are also challenged to provide our medical students with feedback about their physical examination skills. The issues that the authors raised about engaging in optimal timely feedback with students are familiar to our experience in New Zealand.The authors were ambitious to present three aims for this report of their research: 1) to characterize the types of feedback given during the direct observation exercise, 2) to determine student satisfaction with the direct observation exercise, and 3) to compare student satisfaction with the direct observation exercise with small-group learning and traditional lecture formats. I have a few suggestions for the authors to consider to enhance the presentation of their study’s findings.In the methods section, please consider describing the source of small group learning and traditional lecture format data. Please explain why a greater number of students was sampled and reported for the integrated SMN curricular ring and why student data were not matched with the neurology medical student observation feedback questionnaire so the third aim is addressed. It does not seem possible to adequately address the study’s third aim without matched data to made comparisons.At the beginning of the discussion section, please consider stating the key findings of the study in relation to the three aims stated for this article. This statement will help orient the reader to the discussion of the findings’ significance and implications in relation to the other studies discussed in this section. Please consider revising the sentence in the third paragraph that states a correlation was made and that interrater reliability what achieved because only descriptive statistics were used in this study. In the fourth paragraph, please consider enhancing the statement about a clear preference of direct observation versus small group activities without direct observation by conducting statistical tests to establish if there were significant differences among the teaching formats. The use of inferential statistics will also strengthen the claims made in the statements about student satisfaction ratings that pertain to lectures. I appreciate Trudy Roberts’ review comment that challenges the authors to make suggestions to the reader about how to provide ‘bite-size’ learning opportunities to students. I agree with Trevor Gibbs comment that not all of the aims of the study were adequately addressed. I look forward to seeing how this research team implements one of their many interesting ideas for future research.

Reviewer Expertise:

NA

No decision status is available

# Reviewer report: Reviewer response for version 1

**DOI:** 10.21956/mep.19436.r28596

**Published:** 2017-02-04

**Authors:** Trevor Gibbs

**Affiliations:** 1 AMEE

This review has been migrated. The reviewer awarded 3 stars out of 5 An interesting paper to read, covering an important area related to the overall value of a specific type of bedside teaching activity involving feedback. I am not surprised by the results, I would imagine that most students rate the value of bedside teaching on real patients as something they enjoy. It was enlightening to see that the faculty provided some valuable feedback during these teaching sessions. My personal worry is the value that we place upon such low level evaluation findings that lack objective measures. It is good to read that the motivation was realised in the students and that this persisted further into the course- a strongly positive factor, but I think to measure the effects as described in the authors' research objectives needs I feel further research

Reviewer Expertise:

NA

No decision status is available

# Reviewer report: Reviewer response for version 1

**DOI:** 10.21956/mep.19436.r28597

**Published:** 2017-02-03

**Authors:** Trudie Roberts

**Affiliations:** 1 Leeds Institute of Medical Education

This review has been migrated. The reviewer awarded 4 stars out of 5 I enjoyed reading this paper and agree with the authors about the sadly lack of observed feedback of clinical examination skill. Although this paper is useful I would like to pose a challenge to the authors. My clinical colleagues constantly complain of the lack of time to observe students' examination technique in the busy ward round. I would be extremely helpful if they could develop a system of 'bite-size chunks' observation or something along the lines of the 5 minute preceptor model so that on a busy ward round I can ask the student to say examine facial movements or for weakness in a foot. So that the observation can be included in a normal working pattern as well as having to be a scheduled learning event

Reviewer Expertise:

NA

No decision status is available
